# Coding-Complete Genome Sequence of a Yellow Fever Virus Isolated from a Baby Howler Monkey (*Alouatta caraya*) from São Paulo State, Brazil, in 2016

**DOI:** 10.1128/MRA.01244-20

**Published:** 2021-01-07

**Authors:** Márcio Junio Lima Siconelli, Daniel Macedo de Melo Jorge, Luiza Antunes de Castro-Jorge, Benedito Antonio Lopes da Fonseca

**Affiliations:** a Internal Medicine Department, Ribeirão Preto Medical School, University of São Paulo, Ribeirão Preto, São Paulo, Brazil; b Cell and Molecular Biology Department, Ribeirão Preto Medical School, University of São Paulo, Ribeirão Preto, São Paulo, Brazil; Portland State University

## Abstract

We report a coding-complete sequence of a yellow fever virus, strain JabSPM02, containing the 3′ untranslated region and all coding regions. The virus was recovered from an infected howler monkey from a rural area in São Paulo State, Brazil. Our findings show that it belongs to the South America 1E genotype.

## ANNOUNCEMENT

Yellow fever disease (YF) is caused by yellow fever virus (YFV), which is transmitted by mosquito bites in areas of tropical countries in which the disease is endemic. Severe YF is characterized by a mortality rate of approximately 50% (1, 2). In Brazil, there are two known transmission cycles, i.e., an urban cycle (humans to Aedes aegypti to humans) and a sylvatic cycle (nonhuman primates to *Haemagogus*/*Sabethes* spp. to nonhuman primates) (1–3). YFV is an enveloped virus from the *Flavivirus* genus and the *Flaviviridae* family, with a positive-sense, single-stranded RNA of approximately 11 kb (4–7). Since its isolation in 1927 in Ghana (3), only one serotype has been described, but there are seven distinct genotypes, two from the Americas (South America 1 [SA1] and SA2) and five from Africa (West Africa 1 and 2, East Africa 1 and 2, and Angola) (8–10). The SA1 genotype has five characterized lineages (SA1A to SA1E). In Brazil, all recent outbreaks (2008 to 2020) were caused by lineage E (11–15), including the worst epidemic of sylvatic YF in its history (2016 to 2020), with thousands of human and nonhuman primate cases (16).

YFV strain JabSPM02 was isolated, after three passages in Vero cells, from a free-ranging baby howler monkey (*Alouatta caraya*) found in a rural area in the northeast region of São Paulo State, at the beginning of the 2016 epizootic event. RNA from the last cell passage was extracted using a KASVI viral kit (Mobius Life Science, Brazil), and reverse transcription-quantitative PCR (RT-qPCR) (17) was performed according to the manufacturer’s recommendation. cDNA synthesis was performed using the Maxima H Minus cDNA kit (Thermo Fisher Scientific), and Illumina libraries were prepared using the Nextera DNA Flex kit following the manufacturer's recommendation. Sequencing was performed on an Illumina MiSeq instrument using the MiSeq reagent kit v.2 (2 × 150 bp) at LMSeq-UNESP/Faculdade de Ciências Agrárias e Veterinárias (FCAV).

**FIG 1:**
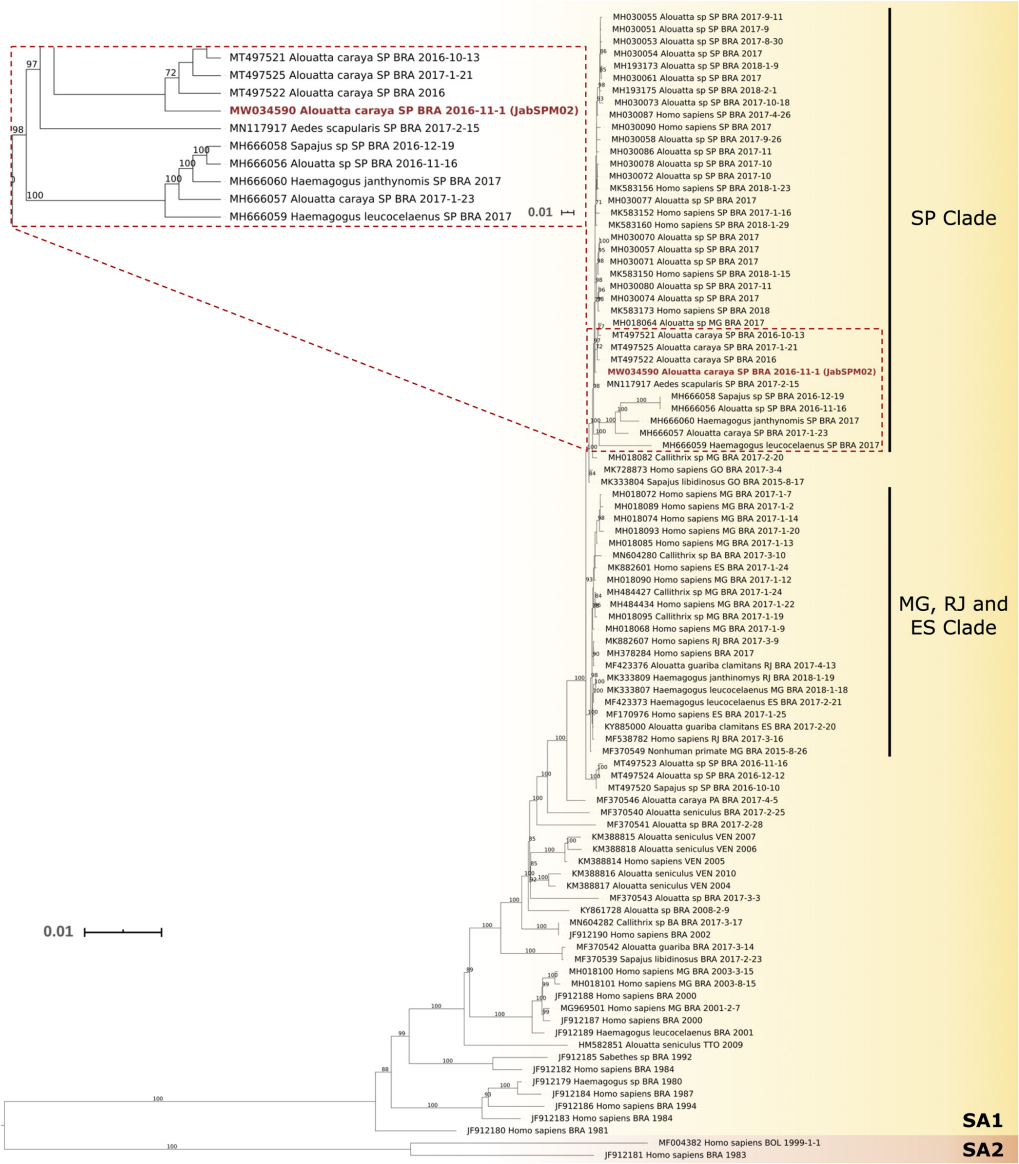
ML phylogenetic reconstruction of YFV coding sequences in South America. Ninety-six sequences from 1981 to 2018 were included in this analysis, presenting 0.01 substitutions per site. The GTR+F+I+G4 substitution model and support analysis of 10,000 replicates were used to obtain the phylogenetic tree. Strains on the yellow background belong to the SA1 genotype and those on the orange background to SA2. The dashed boxes highlight strains from the first epidemic phase in the northeast region of São Paulo State, Brazil; the strain in red is the JabSPM02 strain (GenBank accession number MW034590), which clustered with the SA1 genotype, lineage E, SP clade. Only bootstrap values of ≥70% are shown. YFV 17DD and Asibi_1927 strains were used as outgroups but are not shown.

A total of 202,433 raw reads were quality checked and trimmed using FastQC v.0.11.8, Trimmomatic v.0.3.9, and AfterQC v.0.9.7. Subsequently, the reads were mapped (98.4% of the viral genome using 123,663 reads) against the ES-505 strain sequence (GenBank accession number KY885001) with an average coverage of 1,409.6× using Newbler v.3.0 software (Roche) with default settings, and *de novo* assembly was performed using SPAdes v.3.13.0 with default settings. The JabSPM02 final consensus sequence was 10,945 nucleotides long, with nucleotide and protein identities of 99.75% and 99.39%, respectively, with respect to the ES-505 strain. The final GC content in the assembled sequence was 50.56%. The genome was aligned with MAFFT v.7.0 software using a data set obtained from NCBI. A total of 96 sequences (1981 to 2018) from Brazilian states (São Paulo and Minas Gerais) were selected. The data set was edited and curated for use in the phylogenetic tree to keep the entire open reading frame. The maximum likelihood (ML) tree was constructed using IQ-TREE v.2.0 software with automatic selection, with GTR+F+I+G4 as the best nucleotide substitution model and with support analysis of 10,000 replicates (Fig. 1).

The phylogenetic tree was edited with iTOL v.5. Our sequence clustered with the SA1 genotype, lineage E, São Paulo (SP) clade, which was associated with the first epidemic wave in São Paulo State (18, 19). This virus isolation indicates the occurrence of a sylvatic cycle in the northeast region of São Paulo State at the beginning of 2016, reiterating the importance of continual YFV surveillance to better understand the extra-Amazon transmission cycle in Brazil and to prevent human cases.

The study related to this YFV isolation received the approval of the UNESP/FCAV Ethics Committee on the Use of Animals (process number 010001/17).

### Data availability.

The nucleotide sequence of YFV strain JabSPM02 has been deposited in GenBank under the accession number MW034590; the raw data have been deposited under the accession number PRJNA673883.
